# Bilateral catamenial hemopneumothorax: Diagnostic & management challenges

**DOI:** 10.1016/j.ijscr.2019.07.067

**Published:** 2019-07-31

**Authors:** S. AlAqeel, Y. AlJehani, M. AlMuhaish

**Affiliations:** a National Guard Hospital, Al-Ahsa, P.O. BOX: 3165, Dammam, Saudi Arabia; b King Fahad University Hospital, P.O. BOX 31774, Khobar, 31952, Saudi Arabia

**Keywords:** Catamenial, Hemothorax, Hemopneumothorax, Pneumothorax

## Abstract

**Introduction:**

Catamenial pneumothorax (CP) is a rare and usually miss diagnosed condition that is often characterized by non- specific respiratory or gynecological symptoms. A high index of suspicion is warranted for early diagnosis and management.

**Presentation of case:**

We present a case of 34 year old female who presented with shortness of breath and dyspnea.

**Discussion:**

The radiological diagnosis was pneumothorax with further history, it was apparent that is was a case of primary infertility secondary to possible pelvic endometriosis. She was treated as a case of bilateral catamenial hemopneumothorax after confirming the diagnosis through surgical exploration & repair of right hemidiaphragm.

**Conclusions:**

This case demonstrate the extensive bilateral catamenial hemopnemothorax which is limitedly described in the literature index of suspicion & early diagnosis and management would ensure multiple admission & recurrence.

## Highlights

• TES is becoming more common, with increasing awareness.
• Catamenial pneumothorax represents a complex puzzle, and finding some pieces of the puzzle should suffice for high suspicion on its diagnosis.
• This case demonstrates the variable presentations of TES and the optimal management.

## 1 Introduction

Catamenial pneumothorax (CP) is defined as spontaneous recurrent pneumothorax, occurring in women of reproductive age, in temporal relationship with menses [1]. Endometriosis is a disorder characterized by the growth of endometrial tissue outside the uterine cavity. Usually the ectopic endometrial foci are located in the pelvis, but extra-pelvic disease can be rarely found around 12% [2]. Theories behind that are many: metaplasia theory, embolization theory, retrograde menstruation theory, intraperitoneal air theory and other among such theories. This reflects the poor understanding of such pathology. Thoracic endometriosis is a rare disorder that involves the lungs, pleura, airway, or diaphragm [3]. It could be present as catamenial pneumothorax, catamenial hemothorax, catamenial hemoptysis, lung nodules, cyclic chest pain or very rarely bilateral catamenial hemopneumothorax which we report here in the literature review is scores of such entity. The following case report has been reported in line with SCARE criteria [4].

## 2 Presentation of case

**Fig. 1:**
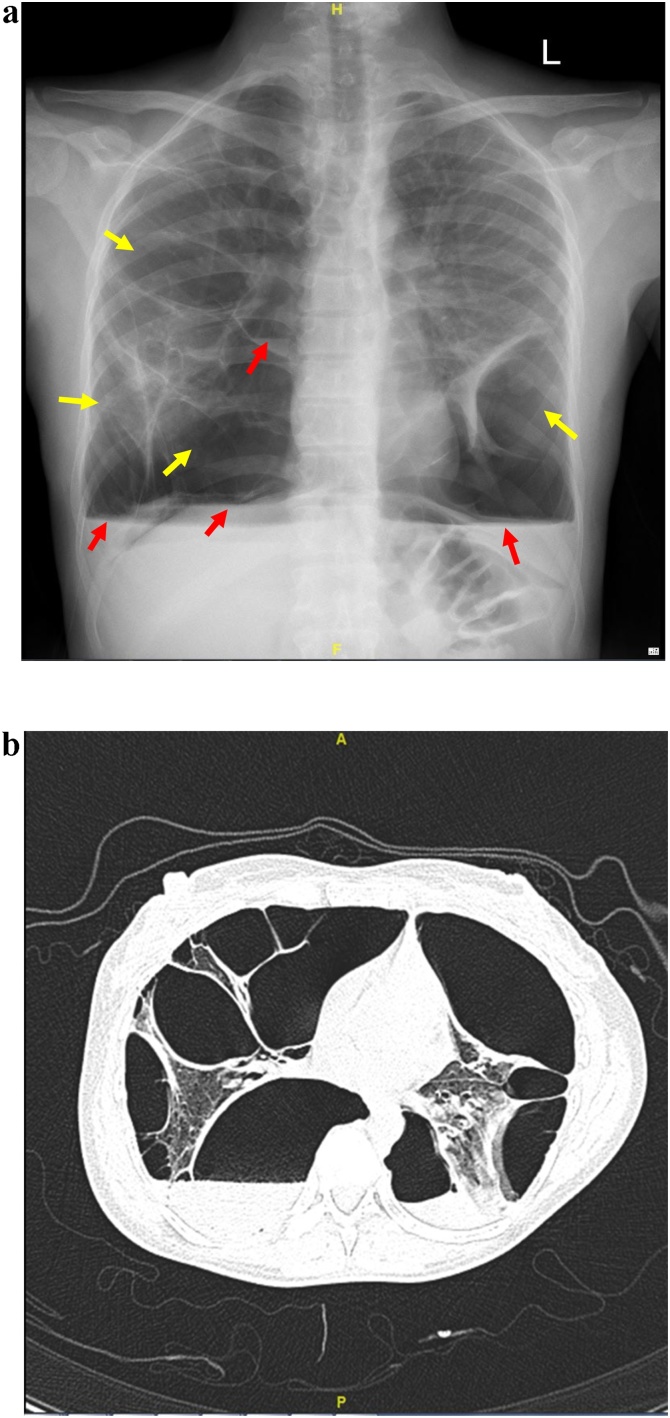
**a**) PA and lateral chest radiograph demonstrate numerous thin walled air containing structures/bullae some shows air fluid level. Yellow arrows: bullae. Red arrows: Air fluid level. **b**) Axial chest CT scan in lung window showing numerous air containing structures/bullae compressing underlying lung parenchyma with some shows air fluid level. No lung nodules. No bronchial wall thickening. No intra-thoracic liver herniation. Yellow arrows: bullae. Red arrows: Air fluid level.

**Fig. 2:**
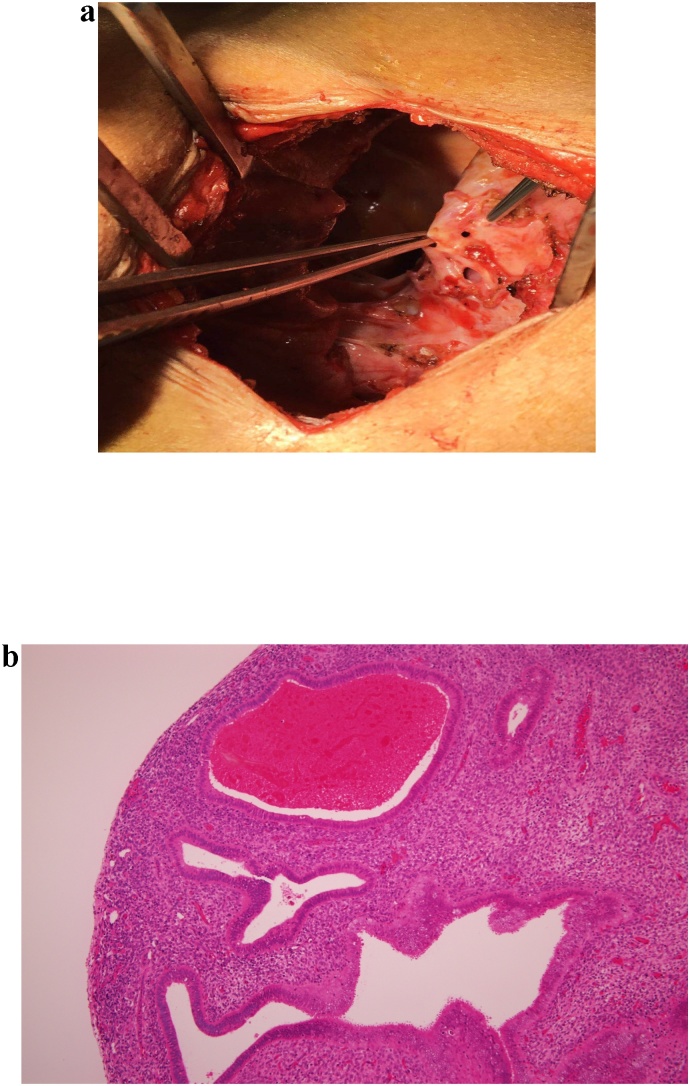
Histopathology.

**Fig. 3:**
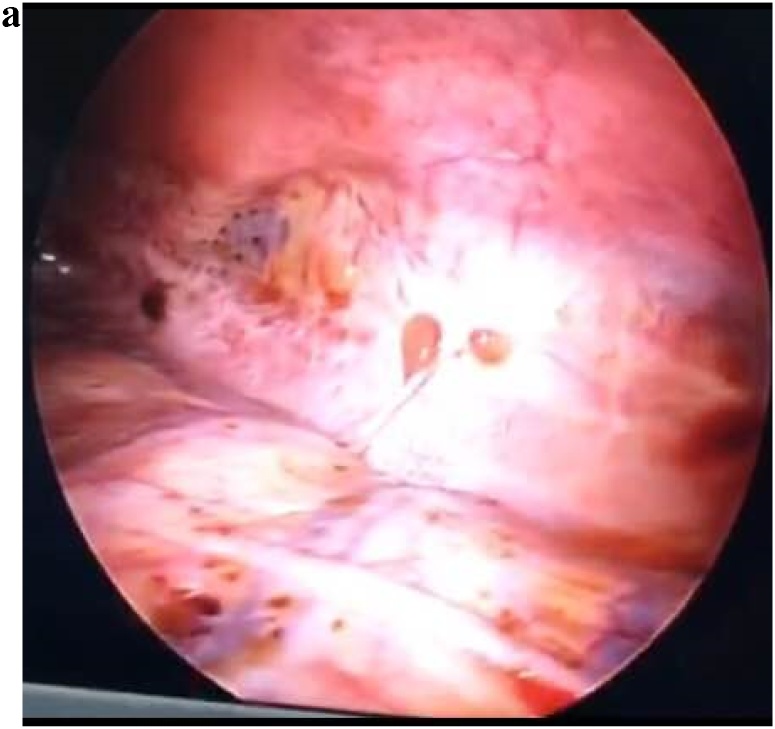
Deposit showed endometrial tissue in VATS exploration.

A 34 year old female who is not known to have any medical illnesses, presented to our emergency room with progressive shortness of breath for several days. She denies any fever, cough, or symptoms of upper respiratory tract infection. There was no chest pain, palpitations or lower limb edema. Her history revealed that she had the same episode years back and diagnosed as a case of right and left primary spontaneous pneumothorax respectively. She was underwent VATS (Video-Assisted Thorocoscopic Surgery) on the left side and tube thoracostomy drainage on the right side. Her medical history demonstrated primary infertility for which she was treated by hormonal therapy but was not successful. The suspicion of pelvic endometriosis was lost in mind but the patient did not complete the treatment. On examination, she was hemodynamically stable her chest examination revealed decreased air entry on both sides with hyper resonant percussion note. Her laboratory findings were within normal expected a picture of microcyctic and hypochromic anemia. Her initial chest x-ray showed bilateral lucencies and reticular patterns (Fig. 1a). Computed tomography (CT) chest demonstrated numerous air containing structures /bullae compressing underlying lung parenchyma with some showing air fluid level (Fig. 1b). A right thoracostomy tube was inserted initially and full lung expansion was achieved, minimal air leak grade 1–2 was observed, the diagnosis was bilateral recurrent pneumothorax initially. A left thoracostomy tube was inserted too and full lung expansion was achieved. The drainage from both thoracostomy tube were serosorgoneous output. Due to high index of suspicion bilateral catamenial hemo-pneumothorax was entertained as a diagnosis. Right VATS exploration and flexible bronchoscopy was done. Upon, exploration, extensive adhesions were encountered, adhesolysis was achieved. The parietal as well as the vesiral pleura showed extensive deposits of varying size (Fig. 2a) the biopsy later on was consistent with endometrial tissue (Fig. 2b) the diaphragm show multiple fenestrations and deposits (Fig. 3a). At this moment, conversion to post lateral thoracotomy was done and the diaphragmatic fenestration were closed primary with prolene 1 sutures. The patient had uneventful postoperative course. She was advised to start on Gonadotropine antagonist but she refused since she is considering fertility. She developed recurrence on the right side few month later and drainage achieved by thoracostomy tube. The drainage was purulent for which exploration and decortication was done. She had uneventful post-operative course. The patient refused any hormonal therapy and that was contributed to her recurrence as far as we think. She agreed on progesterone pills only. She is free of any recurrence for almost years till this report.

## 3 Discussion

Thoracic endometriosis syndrome (TET) remains a commonly missed, or underdiagnosed disease [5]. Increased awareness of this clinical entity and higher index of suspicion should be in any female in her reproductive age presenting with pneumothorax [6]. However, controversies still remain in regards to its pathogenesis. It is believed that some cases of catamenial pneumothorax could be secondary to rupture of either bullae or alveoli following a vascular or bronchiolar constriction or both caused by paracrine secretion of prostaglandin F2-alpha by endometrial implants [7]. In our patient, several endometrial foci were found in the visceral pleura as well as, more interestingly, in the wall of a large and ruptured lung bulla and in the adjacent lung parenchyma. TES can be managed non operatively by medical agents such as danazol, Gonadotropin-releasing hormone (GnHR) agonists, and oral contraceptive pills (OCPs) and operatively by means VATS or standard thoracotomy, in the cases that are refractory to medical treatment [8]. Catamenial pneumothorax is unilateral and right-sided more into almost all cases (85–95%) although there are exceptional reports of left-sided pneumothorax. Bilateral CP is also possible, but it extremely rare. The higher proportion of infertile women among patients with CP/TE might be explained by the frequent association between TE and pelvic endometriosis. The clinical manifestation of CP involves spontaneous pneumothorax preceding or in synchrony with menses, usually presented with pain, dyspnea and cough. Scapular or thoracic pain preceding or in synchrony with menses, history of previous episode of spontaneous pneumothorax, primary or secondary infertility, history of previous uterine surgical procedure or uterine scraping, symptoms of pelvic endometriosis, and rarely history of catamenial haemoptysis or catamenial haemothorax may be present. There are no specific imaging diagnostic criteria. Chest radiography, less often computed tomography (CT), and rarely magnetic resonance imaging (MRI) are performed. Endometriosis has been associated with increased levels of cancer antigen 125, even though it is not a specific marker, but can confirm diagnosis of endometriosis-related pneumothorax. Surgical treatment is the treatment of choice of catamenial pneumothorax and thoracic endometriosis, due to better results mainly less recurrences, in comparison to medical treatment only. Tissue sampling for histologic documentation facilitates the diagnosis, wedged resection along with pleurodesis or pleurectomy has been performed. Hormonal treatment as an adjunct to surgery prevents recurrences of catamenial and/or endometriosis-related pneumothorax. Administration of gonadotrophin-releasing hormone (GnRH) analogue (leading to amenorrhea), in the immediate postoperative period, for 6–12 months is suggested for all patients with proven catamenial and/ or endometriosis-related pneumothorax. Contraindications to hormonal treatment include proven ineffectiveness or significant side effects.

## 4 Conclusions

TES is becoming more and more common, with increasing awareness. Catamenial and/or thoracic endometriosis-related pneumothorax represents a complex puzzle, and finding some pieces of the puzzle should suffice for high suspicion on its diagnosis. This case demonstrates the variable presentations of TES and the optimal management.

## Funding

The following manuscript did not involve any funding resource for any part of the process.

## Ethical approval

Research approval has been attained by the hospital body ethics committee, furthermore consent for writing up the case report while maintaining anonymity was taken from the patient.

## Consent

Written informed consent was obtained from the patient for publication of this case report and accompanying images. A copy of the written consent is available for review by the Editor-In-Chief of this journal on request.

## Author contribution

Writing the paper: Dr. Saja Al-Aqeel.

Data Collection: Dr. Saja Al-Aqeel.

Data Analysis: Dr. Saja Al-Aqeel, Dr. Yasser Al-Jehani.

Literature Research: Dr. Saja Al-Aqeel.

Manuscript Editing and Review: Dr. Yasser Al-Jehani, Dr. Mona Al-Muhaish.

Manuscript Finalization: Dr. Saja Al-Aqeel, Dr. Yasser Al-Jehani.

## Registration of research studies

N/A.

## Guarantor

Dr. Saja Al-Aqeel.

## Provenance and peer review

Not commissioned, externally peer-reviewed.

## Declaration of Competing Interest

The following is a disclosure that the following case report holds no financial or otherwise personal relationship with other people or organizations that may influence our work. No conflict of interest is present.
